# Ultimate figures of merit broadband self-powered obliquely deposited antimony thin film laser detectors

**DOI:** 10.1038/s41598-022-24116-6

**Published:** 2022-11-17

**Authors:** Walid K. Hamoudi, Raid A. Ismail, Munaf R. Ismail

**Affiliations:** 1 Department of Optics Techniques, Al-Farabi University College, Baghdad, Iraq; 2 Department of Applied Sciences, University of Technology, Baghdad, Iraq; 3 Al-Mustafa University College, Baghdad, Iraq

**Keywords:** Materials for devices, Sensors and biosensors

## Abstract

Fabrication of a fast and high detectivity infrared detector operating at room temperature represents a big challenge. Due to the small energy gap of the semiconducting materials used for infrared detectors, the noise becomes considerable factor and the possibility of operating the detector at room temperature is very limited. A study of the figures of merit antimony thin films detector grown by oblique angle deposition technique is presented. Polycrystalline antimony thin films were thermally evaporated on the glass substrates at a angles of 0, 10, 30, and70°. The aim was to develop a wideband (0.649–10.6) µm self-powered laser detectors; operating at room temperature. The deposition angle had a decisive role in the detector specifications, namely, its detectivity, responsivity, linearity, and response time. At θ = 70° deposition angle; maximum detectivity and fastest response were achieved. The variation of rise time with deposition angle was linear, and the rise time was around 50 ns at 70°. The antimony detectors showed about the same specific detectivity ~ 10^9^ Jones at 300 k for the wavelength range of 1.064–10.6 µm.

## Introduction

The main factors affecting thin film growth are the type of substrate, its temperature, the vaporization temperature, the angle at which the vapor is deposited, the pressure of contaminating gases, the vacuum pressure, and the rate of deposition^1^. When the substrate is placed parallel to the evaporant, a uniform thin layer is produced. However, when the substrate is tilted, surface shadowing is created, which creates nano-columnar growth structures with wide pores between them^2^. The surface area is increased and anisotropic optical, magnetic, and electrical characteristics are produced by the oblique deposition of thin films. The chemical makeup of the material being used determines the oblique angle of the nano-columns. The trajectories of species in the vapor phase close to the surface rely primarily on their relative velocities, chemical composition, and distance from the surface^3^. Due to its semi-metallic nature and extremely isotropic Fermi surface, Sb is being studied as an important thermoelectric candidate^4–7^. It has applications in pulsed laser detectors due to its low thermal capacity and quick response time. Studies on Sb films' electrical resistivity and thermoelectric power produced significant discrepancies, mostly as a result of the deposition conditions^8^. The density of the thin film decreases with increasing angle, and the grain growth can exhibit a structure with high density rods or needles separated by low density gaps^9,10^; where thin film density decreases with increasing angle^11,12^. Geometrical shadowing prevents the deposition of particles in regions existed behind initially formed nuclei and produces tilted columnar and highly porous microstructures. The first nuclei formed during the earliest deposition stages creates a shadow behind, which stops any further evaporated material within these ”shadowed” regions. As deposition goes on, these nuclei induce tilted and separated nanocolumns that give rise to porosity, birefringence and magnetic anisotropy^13^. The direction of columnar development often forms an angle (β) with the substrate's normal, and often (β < θ); where θ is the deposition angle. The correlation between these two angles is^14^:

1 $${\text{2 tan }}\upbeta  = {\text{ tan }}\uptheta$$

As the angle is increased during oblique deposition, the asymmetry, surface area, and column separation all rise. A thin film's potential gradient (dV/dx) results from a temperature gradient (dT/dx). The ratio of e.m.f. (∆V) per temperature differential (∆T) across the opposite sides of a thin film is the thermoelectric power given as:

2 $$ S = - \frac{{\Delta {\text{V}}}}{{\Delta {\text{T}}}} $$

In the phenomena of electric conduction and thermoelectric action in metals, the cooperation of electrons is in two conditions. The conduction mechanism of free electrons in the film: those passing from atom to atom quickly and others between the atoms which act according to gas laws, affects the thermoelectric behavior of thin films. The thermoelectric power (S) for metallic films is given as^15^:

3 $$ S = \left( {\pi K_{B}^{2} T^{3} e} \right) \left( {\frac{1}{\sigma }\frac{d\sigma }{{dE}}} \right)_{{E = E_{r} }} $$

where σ is the electrical conductivity of the film, E and E_F_ are electron and Fermi energies, respectively, T is the temperature, e is the absolute value of electronic charge, and k_B_ is Boltzmann constant. When placed obliquely, thin films of some materials show potential variation along their length. Anisotropic stresses are produced in these films when exposed to laser pulses; therefore generating an e.m.f. The anisotropic thermoelectric properties of Sb film comes from structural asymmetries, for example, the different properties along the crystallographic directions Theses stresses are due to both piezoelectric and thermoelectric effects. Piezoelectric effect dominates when using nanosecond (Nd: YAG and CO_2_) laser pulses due to the ultrasound waves generation. The thermoelectric effect, on other hand, is the larger effect when microsecond (Ruby) laser pulses were used. The film resistance and, consequently, the generated e.m.f are significantly influenced by the deposition angle^16^. Conduction electrons can go farther in crystalline media than ion core separation without scattering. Thermal motion is mostly responsible for this Ohmic resistance. Conduction electrons are approximated in the Drude model by the free electron gas. The electric field strength, E, and current density, J, are used to express Ohm's law as follows:

4 $$ J = \sigma E $$

Conduction electrons are slightly scattered (glancing collision) by vibrations or imperfections. Such collisions follow one another and have the same overall impact as a single collision. The relaxation time, τ, is the typical interval between two collisions (of the same electron). As τ rises, an electron's drift velocity (v_d_) falls off exponentially. Because the electron drift velocity is lower than the thermal average velocity, v_th_, of the individual electrons, the current diminishes. The mean free path, λ, is the average distance between two scattering collisions (caused by flaws and thermal fluctuations) that the electrons travel at while moving with the thermal velocity.

5 $$ \lambda = v_{F} \tau $$

where ʋ_F_ is the Fermi speed

6 $$ v_{F} = \left( {2E_{F} /m_{e} } \right)^{0.5} $$

Under the application of a field, E, electrons experience a force F = ma. For an electron emerging from a collision with velocity v_o_, the velocity after time t is given by:

7 $$ v = v_{o} - \frac{eEt}{{m_{e} }} $$

If the electrons are scattered randomly by each collision, v_o_ will be zero. If the time is equal to the scattering time, the drift velocity will be:

8 $$ v = \frac{ - eE\tau }{{m_{e} }} $$

For **n** free electrons per unit volume, the current density J is:

9 $$ J = - nev $$

10 $$ J = \frac{{ne^{2} \tau }}{{m_{e} }} $$

The conductivity σ = n e μ, where μ is the mobility, which is defined as

11 $$ \mu = \frac{{\text{v}}}{E} = \frac{eE\tau }{{m_{e} E}} = \frac{e\tau }{{m_{e} }} $$

With no applied field, the electrons move around randomly. The deposition angle and substrate temperature had a significant impact on these films' sensitivity as well as other aspects of their properties. In tilted La_0.5_Sr_0.5_CoO_3_ thin films produced on vicinal cut LaAlO_3_ (100) substrates, the transverse laser induced thermoelectric voltage effect has been studied when films are irradiated by laser pulses at room temperature. The laser-induced thermoelectric voltage effect was assumed to be caused by the transverse Seebeck effect of obliquely deposited thin films: When a thin film is heated by laser light, a temperature gradient perpendicular to the film surface is created. The Seebeck effect produces a voltage from this temperature gradient. The voltage dependence on film thickness exhibits an increase followed by a decline^17^. Self-powered stable Sb-based detector was constructed. This detector was capable of detecting laser wavelengths in the range of 0.405–1.064 µm^18^. To the best of our knowledge, no information is available on how the Sb deposition angle affects the effectiveness of laser detection. The goal of this research work is to find out how well obliquely formed Sb thin films can detect visible, near-IR and mid-IR radiations.

## Physical mechanism and working principle of the detector

When a high laser energy pulse is incident on the obliquely deposited Sb thin film, a transverse voltage (∆V) is generated due to the temperature change (∆T) originating from the columnar structure. The latter is created from oblique deposition. The transverse voltage is proportional with the mechanical stresses that originates from the incident laser pulse and proportional to the Sb detector dimensions. ∆V depends on the Sb Seebeck coefficient which improved in oblique deposition. Due to the columnar growth along the y-axis, the Sb detector length was chosen to be much larger than its width. The small energy-gap of the antimony facilitated the detection of longer wavelengths (10.6 µ).

## Experiment

**Figure 1:**
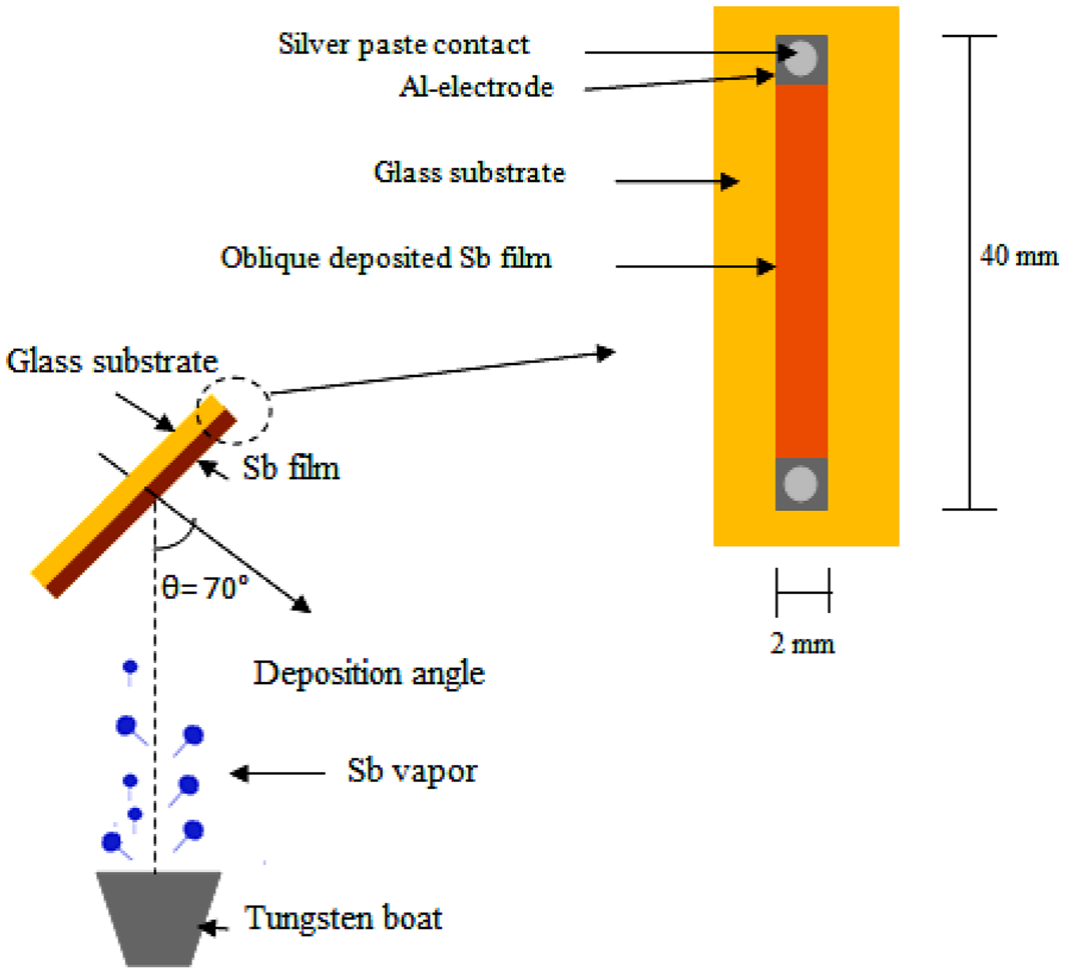
Oblique angle deposition set-up with configuration of Sb thin film detector.

High optical quality glass substrates were first cleaned with alcohol, followed by 15 s of HCl acid cleaning, rinsing them afterward in deionized water, and then a final 10 min of alcohol cleaning in an ultrasonic bath. Thin antimony films with a purity of 99.99% were thermally evaporated on glass substrates at various angles (30–80°). Using an X-ray diffractometer (XRD-6000, Shimadzu) with CuKα source, the structure of Sb film deposited at different orientations was examined. Using a scanning electron microscope (T-scan Vega III Czech), the film's surface morphology was examined. The detector, which measures 40 mm in length and 2 mm in width, was fabricated by obliquely depositing Sb thin film on a glass substrate using a thin metal mask that was specially constructed for the task. With a deposition rate of (5 nm/s), the vacuum pressure was dropped to 10^–6^ mbar, allowing for the deposition of films of various thicknesses. The schematic diagram for oblique angle deposition is shown in Fig. 1, where θ is the angle of incidence between the Sb vapor stream and the glass substrate's normal. Ohmic contacts were then formed by evaporating a high purity rectangular shape aluminum thin film on the two sides of the Sb film. A silver paste contact was employed following the deposition of ohmic contacts. The Seebeck coefficient of Sb film was measured as a function of deposition angle. It was estimated from the slope of the Seebeck voltage (ΔV) versus the applied temperature difference (ΔT). One film side was heated and the other was cooled by heat sink. By using K-type thermocouple, the temperature difference was measured. These measurements were repeated twice to obtain an average value. All electronic and optoelectronic measurements were made in this work at room temperature and in ambient conditions. To evaluate the figures of merit of the antimony thin film detectors; Ruby, Nd: YAG and CO_2_ pulsed lasers with the specifications shown in Table 1 were used. A storage digital oscilloscope with a bandwidth of 250 MHz was used to assess the voltage responsiveness, detectivity, and rise time of the photodetector.

**Table 1:** The main parameters of the lasers used.

Laser type	Wavelength (µm)	Pulse duration	Repetition rate (Hz)	Spot size (mm)
Ruby	0.649	100 µs	2	2
Nd:YAG	1.064	450 µs	2	2
TEA CO_2_	10.6	200 ns	1	10

## Results and discussion

**Figure 2:**
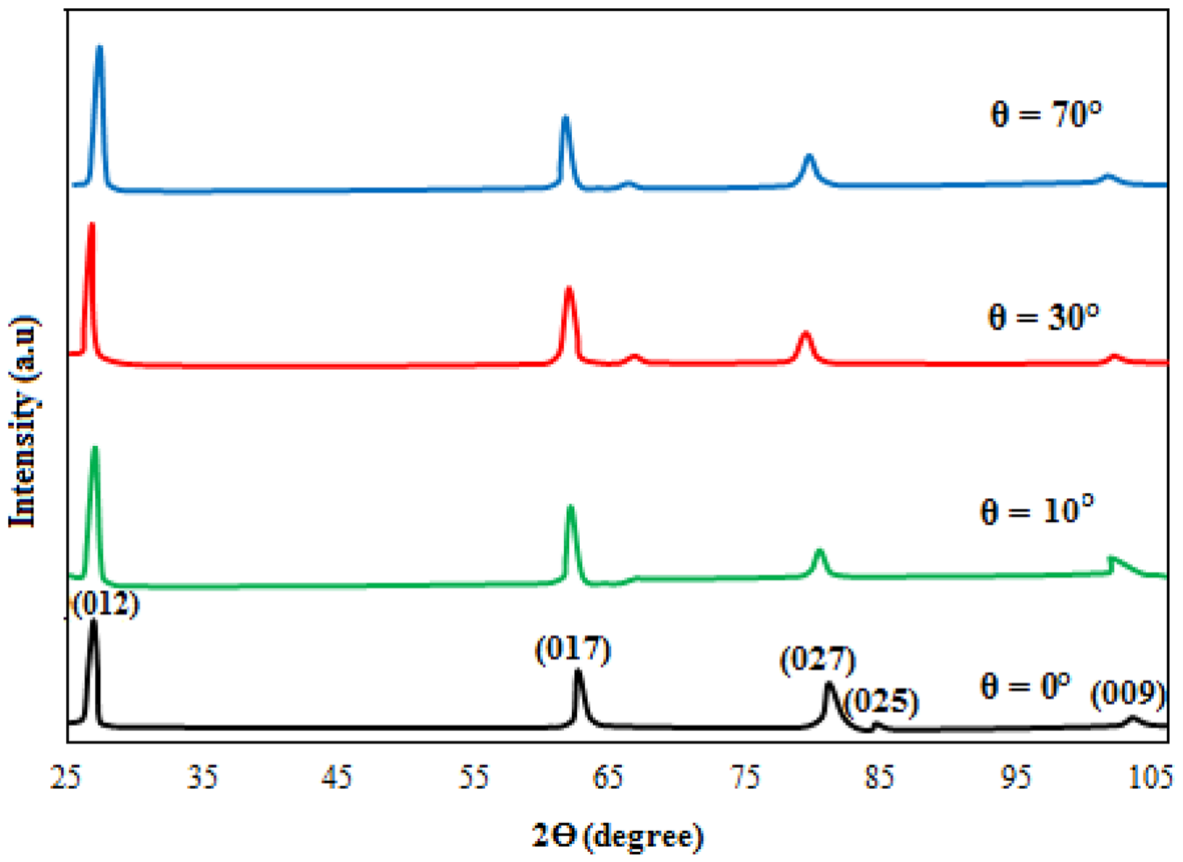
XRD patterns of antimony films deposited at θ = 0, 10, 30, and 70°.

The XRD patterns of Sb films deposited at 0, 10, and 70° are shown in Fig. 2. At θ = 0°. The film exhibited five peaks observed at 27.2, 61.6, 79.5, 84.6, and 102°, which correspond to (012), (107), (027), (025), and (009) planes, respectively. All the observed peaks are indexed to Sb with rhombohedral structure (space group R3m) according to JCPDS # 01-085-1324)^19^. At θ = 30°, there are five peaks located at 27.2, 61.6, 66.2, 84.6, and 102°, which belong to (012), (107), (122), (025), and (009) planes, respectively. A new peak has appeared at 66.2° and the peak at 79.5° has disappeared. Increasing the deposition angle to θ = 70°, similar peaks to those at 30° have appeared; indicating a more or less similar growth mechanism of tilt angles. At oblique deposition, the intensity of the strong peak in the (012) plane direction increases and sharpens; reflecting an improvement in the film's crystallinity. The XRD pattern showed no new peaks associated with other antimony phases or impurities; i.e., there is no phase transformation in the obliquely deposited film. The crystallite size, strain, and dislocation density in the (012) plane were determined from XRD analysis and tabulated in Table 2. The following Scherrer equation was utilized to estimate the crystallite size which increased with the deposition angle; due to the formation of elongated grains with columnar growth.

12 $$ D = \frac{0.9\lambda }{{\beta \cos \Theta }} $$

where λ is the X-ray source wavelength (CuKα, λ = 1.5406 Â), and β is the full width at half maximum FWHM of XRD peak. The oblique deposition has created vertical and diagonal stresses and structural defects along the film as a result of the presence of holes, which in turn produced dislocation defects.

**Table 2:** Average grain size along (012) plane strain (ε), and dislocation density (δ).

Deposition angle	FWHM (deg)	D (nm)	δ (nm)^−2^ × 10^–2^	ε × 10^–2^
0°	0.305	25.27	0.156	0.1298
10°	0.303	25	0.159	0.1300
30°	0.309	24.95	0.161	0.1312
70°	0.382	20.18	0.245	0.1620

**Figure 3:**
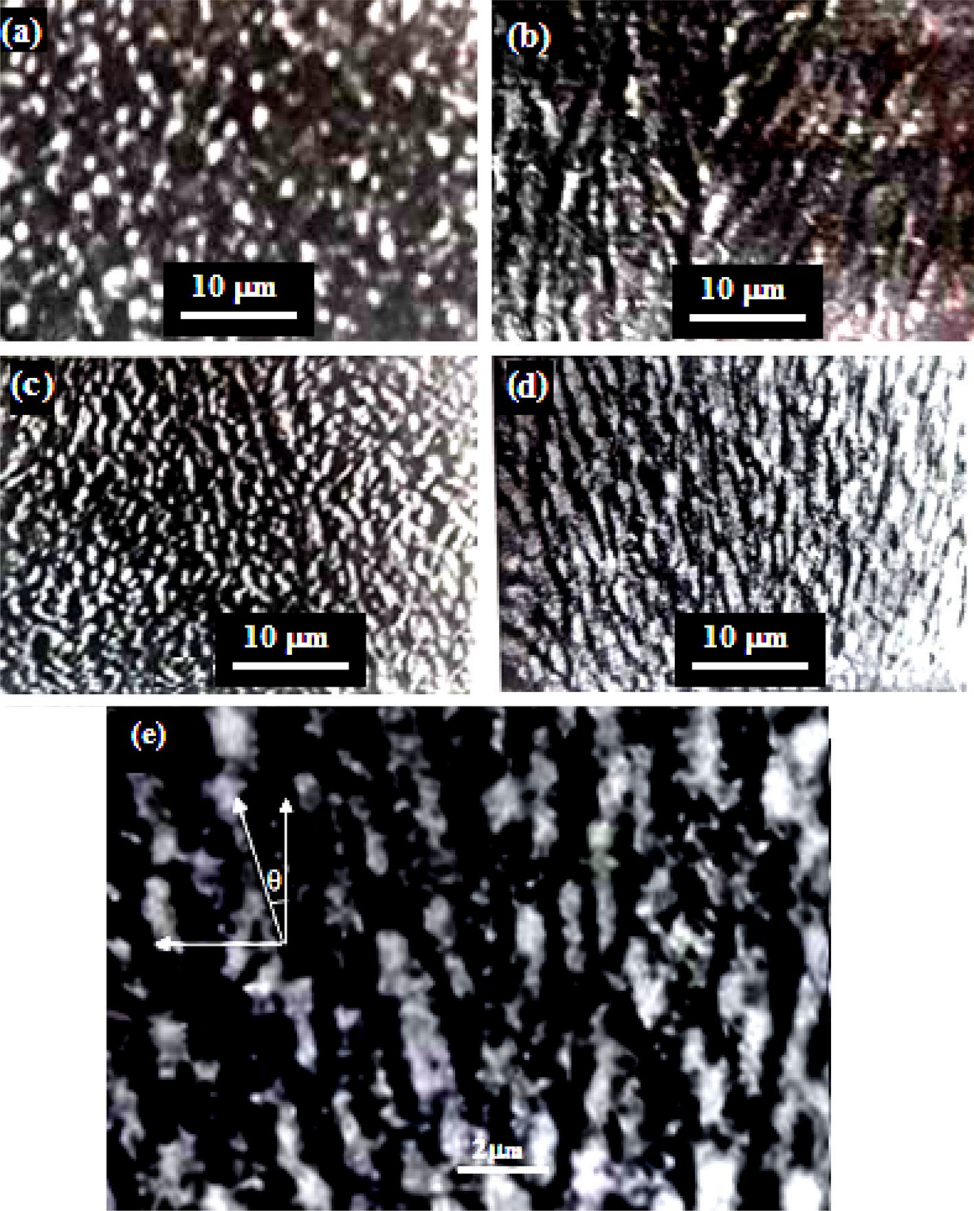
SEM images of antimony films deposited at (**a**) θ = 0°, (**b**) 10°, (**c**) 30°, (**d**) 70°, and (**e**) shows the tilted grains of the film deposited at 70°.

The SEM images of antimony film deposited at angles of = 0, 10, 30, and 70° are shown in Fig. 3, with the morphology drastically altering with deposition angle. With increasing tilt angle, the columnar grain concentration that is dispersed across the film surface also rises. Only spherical grains with an average size of 2 µm are visible in the SEM image of the Sb film at = 0°, and no voids or micro-cracks were observed on the film surface. It is very clear that the SEM image of the film deposited at 70° shows the existence of tilted grains ~ 22° (Fig. 3e) and holes between the grains that help form a hydrophobic surface due to the shadowing effect^20^.

**Figure 4:**
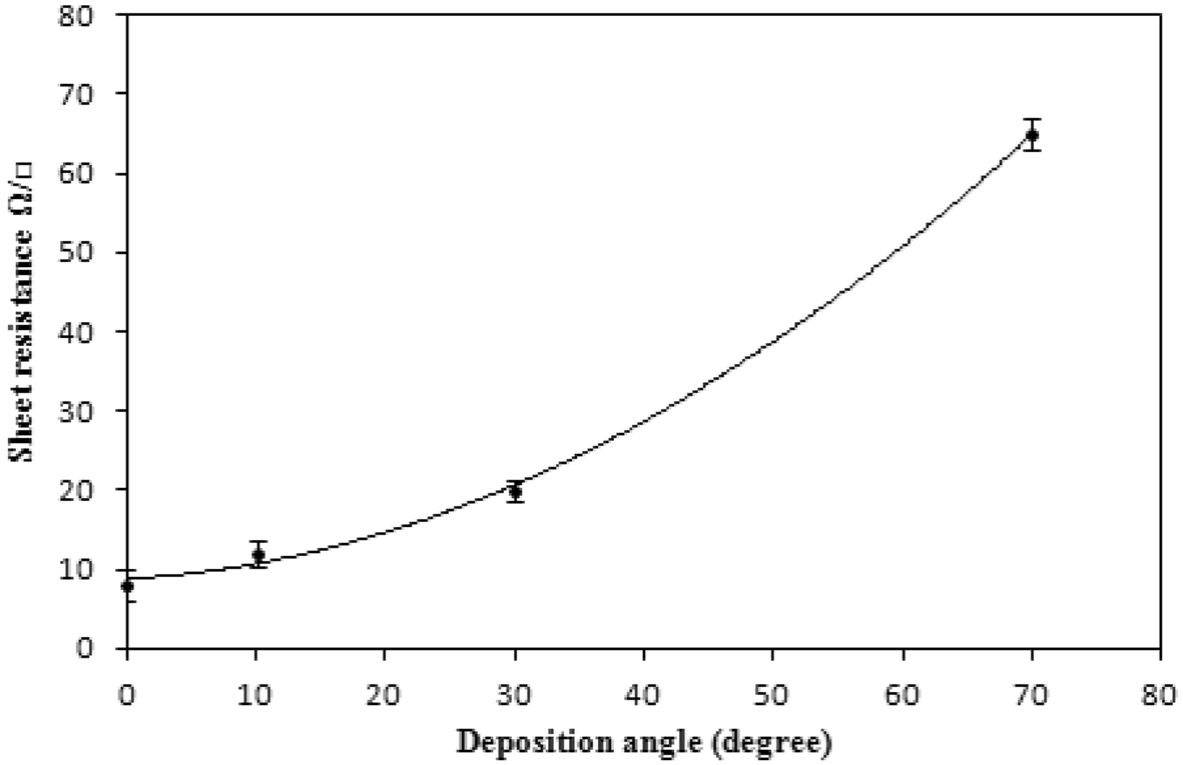
Dependence of film sheet resistance on the deposition angle.

The effect of the deposition angle on the sheet resistance of antimony films is depicted in Fig. 4. It is increased as the angle of deposition increases. This result arises from the formation of columnar growth and elongated grains, which lead to the formation of grain boundaries. In addition to decreasing the thickness, the elevation of deposition angles showed an increased sheet resistance of the antimony films, as depicted in Fig. 4. This arises because of columnar growth and the formation of elongated grains that create grain boundaries.

**Figure 5:**
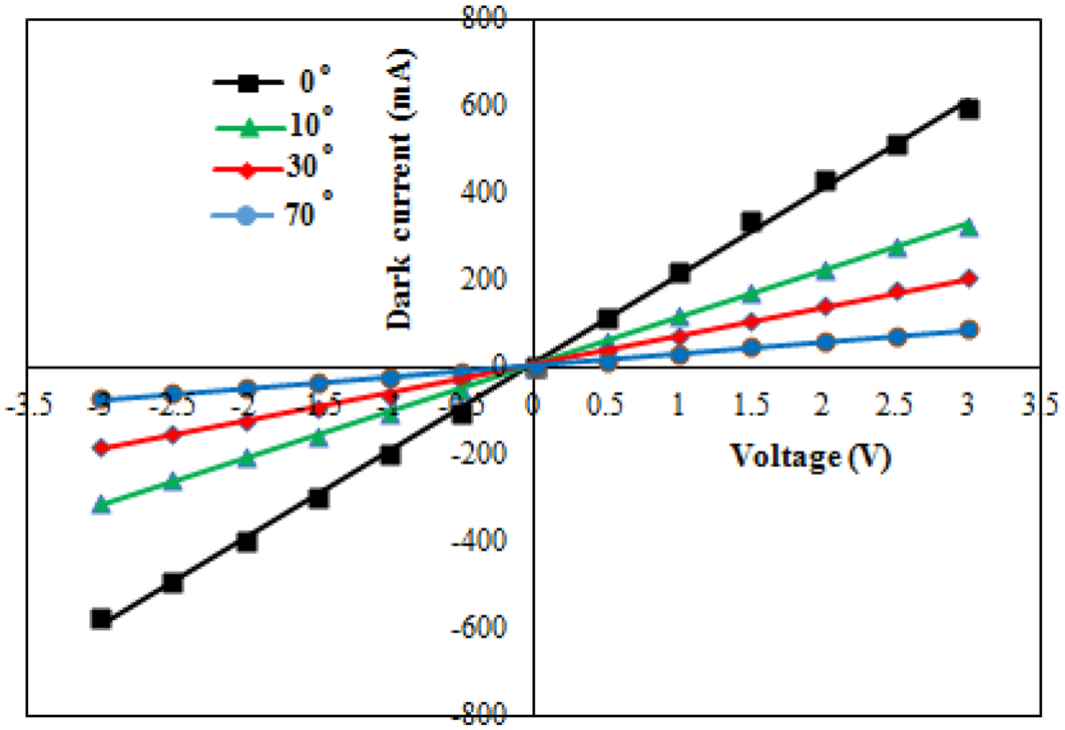
Dark I-V characteristics of Al/Sb contact deposited at different deposition angles.

Figure 5 displays the forward and reverse I–V characteristics of the Al/Sb film contact and demonstrates a linear Ohmic connection.

**Figure 6:**
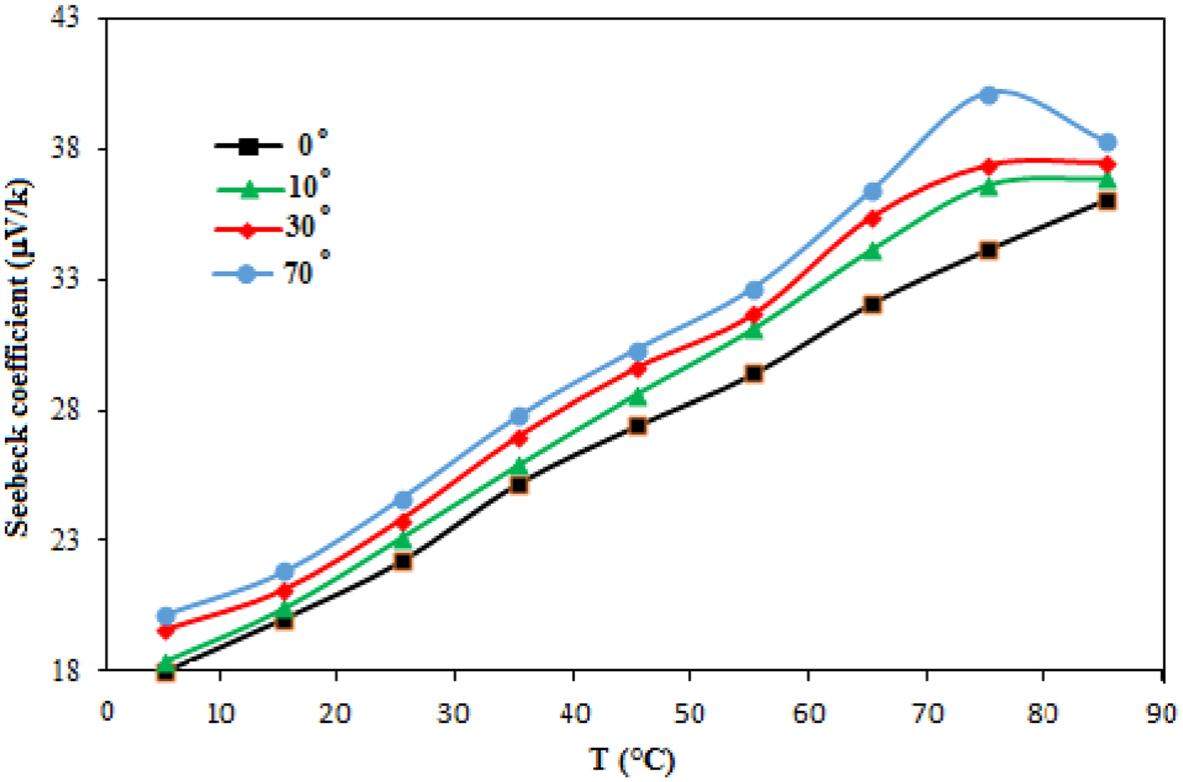
Seebeck coefficient of Sb deposited with operating temperature.

Due to the high film electrical resistance, the current decreases as the deposition angle increases. This can be attributed to the decreasing grain size and the rise of grain boundary. The variation of Seebeck coefficient with temperature of Sb film deposited at various deposition angles is shown in Fig. 6. The thermoelectric tensor comprises of two parts:${S}_{\parallel}$ along the major axis of trigonal symmetry, and ${S}_{\perp }$, along a binary axis that is orthogonal to the trigonal axis^21^. All S values are positive and the film deposited at 70° has the highest value due to its high electrical resistance (Fig. 4). The semimetal antimony film was selected as a thermoelectric material because of its high Seebeck coefficient and low conductivity which maximize the figures of merit ZT of the thermoelectric material according to the following equation

13 $$ ZT = \frac{{TS^{2} \sigma }}{{k_{e} + k_{l} }} $$

where T is temperature, S is Seebeck coefficient, σ is the electrical conductivity, k_e_ is the electronic thermal conductivity, and k_l_ is the lattice thermal conductivity. The value of ZT was calculated with aid of Eq. 5 together with the results of Seebeck and electrical resistivity and was found to be 1.3, 1.18, 0.27, 0.16 for deposition angle of 0, 10, 30, and 70°, respectively. This indicates that thermoelectric properties improve with deposition angle.

**Figure 7:**
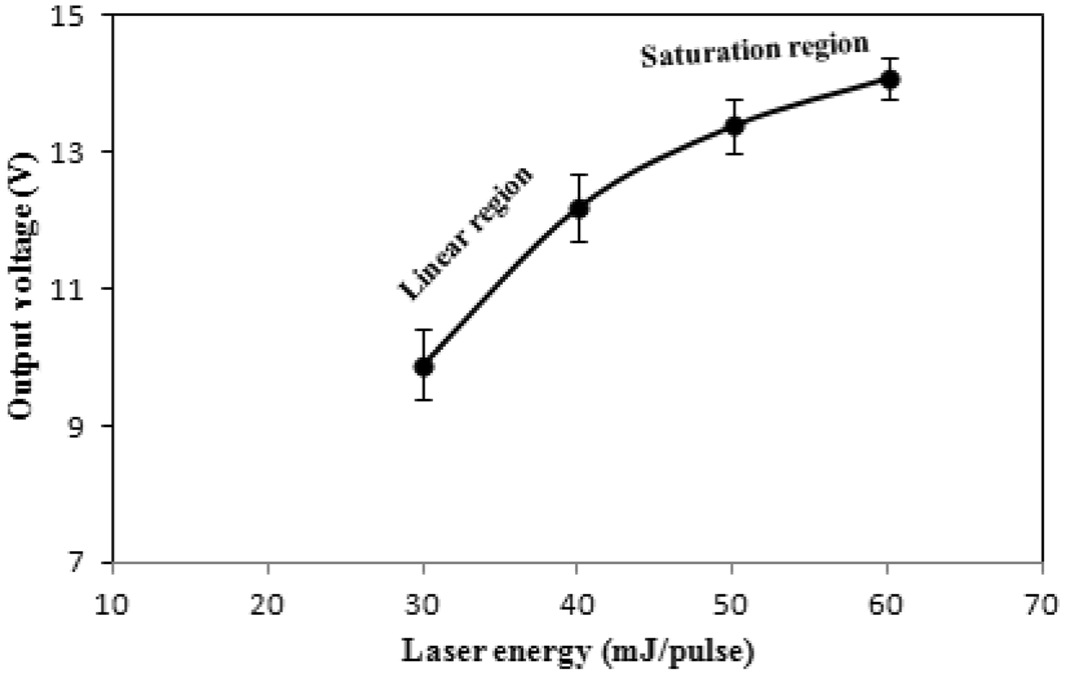
Variation of output voltage with pulse laser energy at zero bias for an Sb detector deposited at θ = 70°.

**Figure 8:**
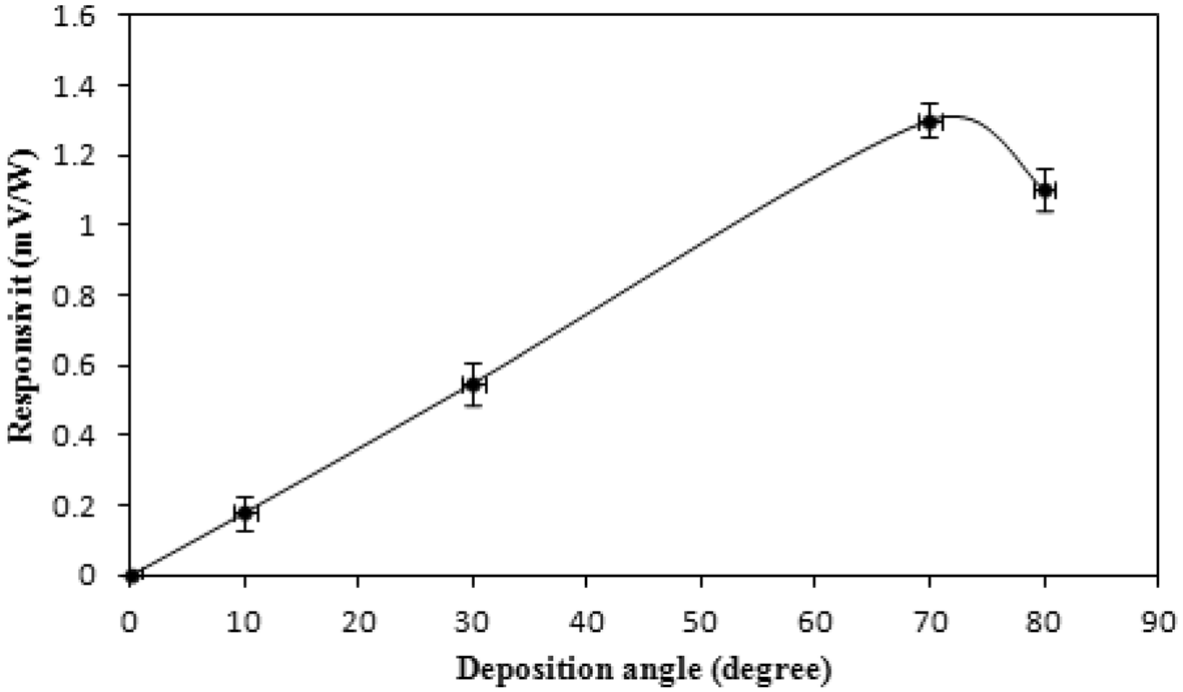
Responsivity of the Sb detector for ruby laser as a function of deposition angle.

The detectors' linearity characteristics of the 1.064 m Nd: YAG laser are shown in Fig. 7. There are two distinct regions: the helpful usable range, where the output signal is proportional linearly to the input laser energy up to 40 mJ, and the undesirable region; where the detector tends to saturate. The responsivity curve for the ruby laser pulses is shown in Fig. 8 at various deposition angles. The link between voltage and responsiveness is as follows:

14 $$ R = \frac{V}{P} $$

where P is the laser power and V is the produced voltage from the detector. This figure depicts a maximum voltage responsivity of (1.5 mV/W) at a 70° deposition angle and 300 nm film's thickness. It shows a linear responsivity behavior with deposition angle up to 60°, after which the responsivity starts to sharply increase until reaching a maximum at 70°. All antimony thin film detectors, under investigation, exhibited this behavior consistently.

**Figure 9:**
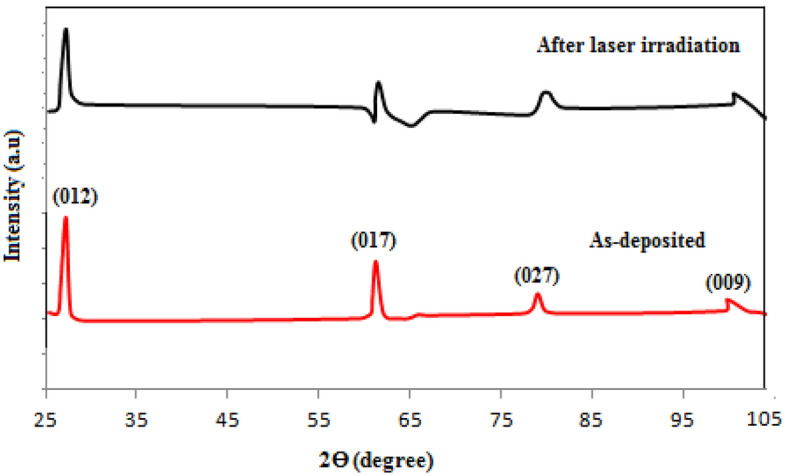
XRD patterns of antimony film deposited at θ = 70° before and after Ruby laser irradiation.

When atoms are positioned asymmetrically during oblique deposition, mechanical stresses are produced that increase linearly with the deposition angle. Similar to the flaws created in the junction region of heterojunction semiconductor detectors, this makes the thin film more responsive to incident laser pulses. Ruby laser irradiation of 1 kW power didn’t cause any physical damage or saturation behavior in the antimony detectors; indicating the capability of these detectors to function at high laser power levels without using attenuating optical elements and also their potential for application in laser energy meters. To confirm this fact, Fig. 9 shows the XRD of antimony film deposited at θ = 70° before and after laser ruby irradiation. The XRD pattern at which the laser irradiated film is still crystalline without significant decrease in intensity of the XRD peaks indicates a nearly stable film’s crystallinity.

**Figure 10:**
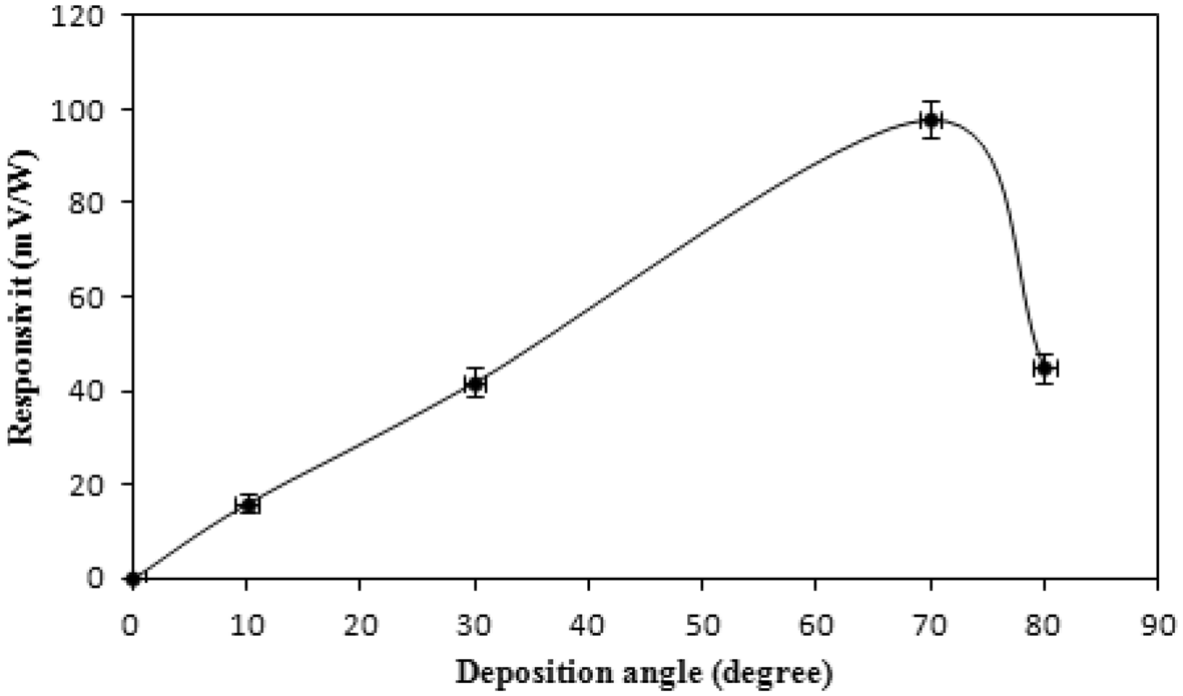
Responsivity of Sb detector prepared at different angles for Nd: YAG laser pulses without using bias.

The output ruby laser pulse was photographed by utilizing antimony thin film detectors deposited at a 70° angle and at room temperature, as shown in supplementary information (Fig. S1). In Fig. 10, the responsivity of antimony thin film detectors prepared at various deposition angles to Nd: YAG laser pulses is depicted.

All of these detectors exhibit an increase in responsiveness with increasing deposition angle, peaking at an angle of 70°. The responsiveness decreased as the deposition angle was increased over 70°, reaching roughly 60% of its maximum values at 80°. The Exfoliation and uneven longitudinal growth of the film, significant air interference and shadowing effect are only a few reasons of this behavior at large deposition angle which could cause irregularities and high concentration of pores and a decrease in responsivity^22^. As revealed in supplementary information (Figs. S2 and S3) illustrate the Nd: YAG laser pulses that the antimony detectors recorded for two different thicknesses and for two different laser energy pulses; with 80 mV/W responsivity achieved at 300 nm thicknesses. The output signal linearity from antimony detectors for various thicknesses, evaluated with Nd: YAG laser (supplementary information Fig. S2), is presented in Table 3. The current antimony detectors, in contrast to semiconductor detectors, have output voltages that are strongly dependent on the deposition angles, and are crucial for the development of columnar structure.

**Table 3:** Linear response of different thicknesses Sb detectors to Nd: YAG laser.

Thin film thickness (nm)	Output signal (V)
100	8
200	8
300	8
400	8.4

**Figure 11:**
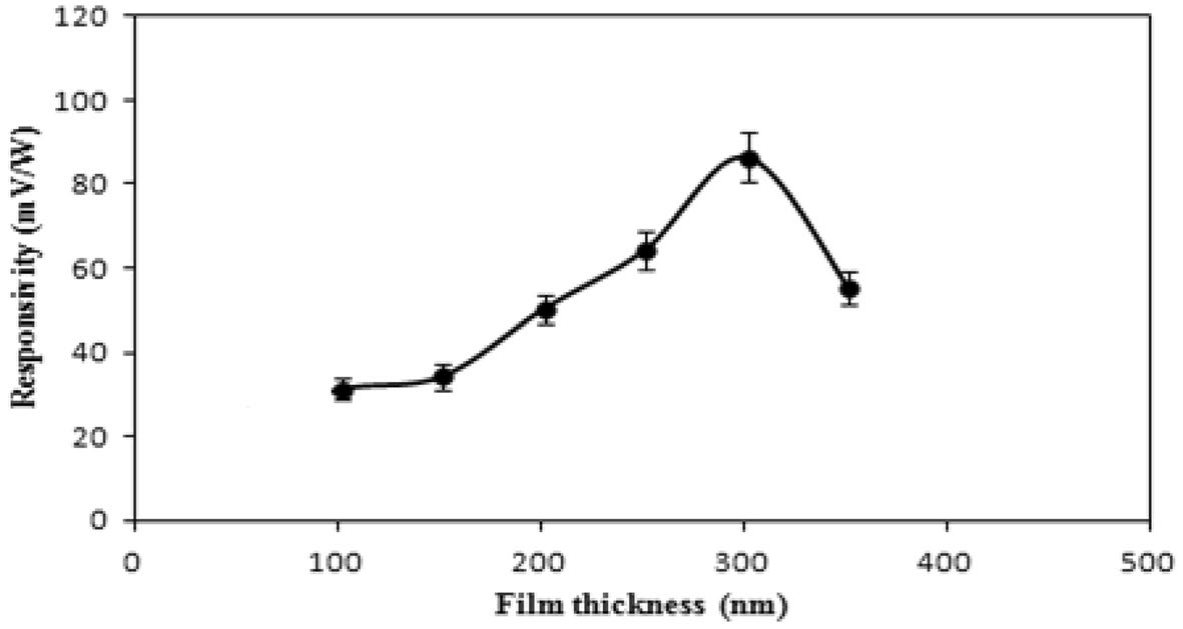
Responsivity of Sb detector for Nd: YAG laser pulse as a function of film thickness.

**Figure 12:**
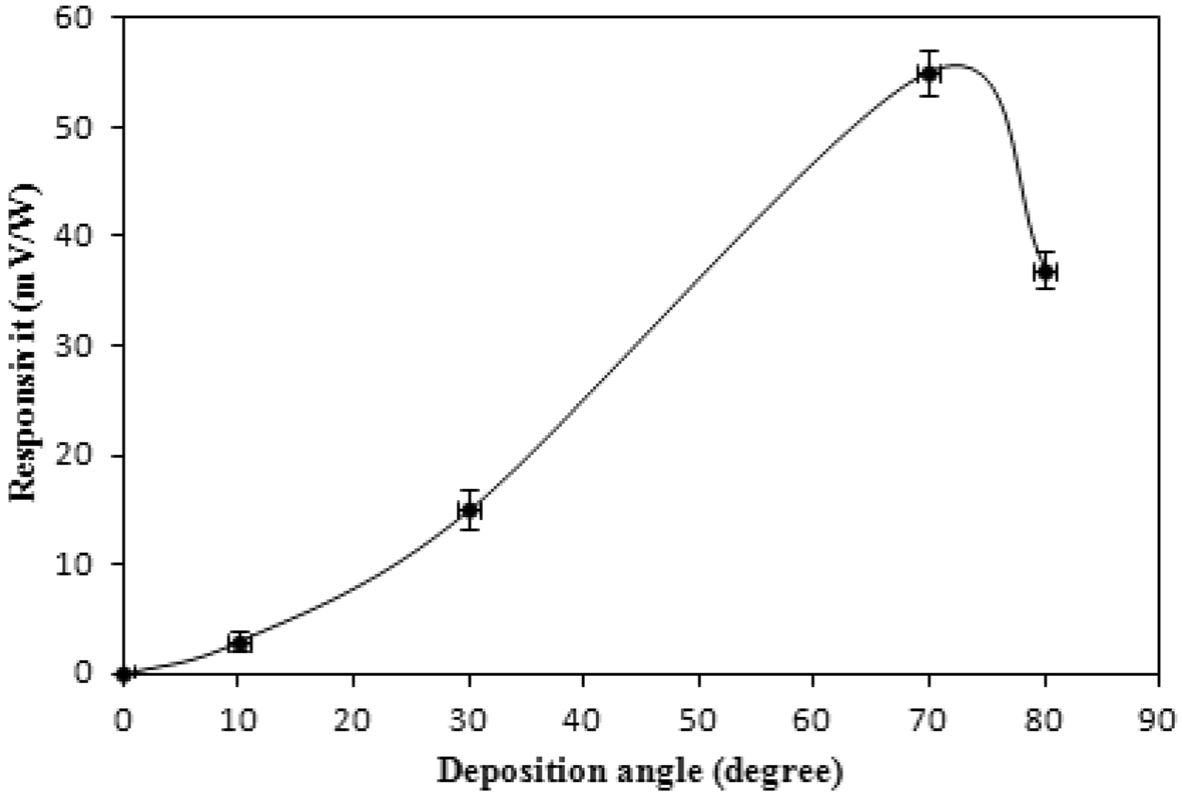
Responsivity versus deposition angle behavior of Sb detectors; irradiated with CO_2_ laser pulses.

The responsivity has been reduced to almost half its maximum value by thicker layers. Response is influenced by the antimony film's thickness (d), which is correlated with the film's absorption coefficient (α). Only a small portion of incident light is absorbed for a thickness of d ≤ α^−1^, which results in poorer responsiveness. As the thickness is increased, the responsiveness steadily develops until it reaches its maximum. The surface states and structural flaws that result in a decrease in the thermoelectric coefficient, or a reduction in the temperature rise ∆T caused by laser heating, will be enhanced by further increases in thickness^23^. Figure 11 shows the responsivity of the detector for Nd: YAG laser pulse as a function of film thickness when utilizing 50% optical attenuator. With respect to Table 3, the constant values of output signals (voltage) indicate a saturation of the detector. This has neccitated the insertion of 50% optical filter. Figure 11 reflects the real responsivity values without saturation. Figure 12 exhibits the responsivity-deposition angle curve for antimony thin film detectors irradiated by a CO_2_ laser pulse.

As mentioned before, a maximum responsivity was attained at a thickness and deposition angle of 300 nm and 70°, respectively. Storing the antimony detectors in the laboratory, at 30 °C and 50% humidity, didn’t show any aging effect, with about a 3% responsivity decrease after six months. The low wettability was also another advantage of these detectors. One antimony detector was immersed in water and used immediately to capture laser pulses. The responsivity of the photodetector wasn’t affected at all. We believe that the angle of deposition of 70° is greater than the critical angle at which water droplets adhere to the detector surface. Table 4 presents the increasing output signal with deposition angle when illuminating the antimony detectors with TEA-CO_2_ laser pulses. The output signal of the detector at 70° deposition that is illuminated with a CO_2_ laser pulse is shown in supplementary information (Fig. S4). As shown in supplementary information (Fig. S4), the decay time (from 90 to 10% points) is longer than the rise time. The tail is due to the mismatch resistance (open load) that is used in order to get higher voltage signal as well due to surface states and traps. For single shot applications^24–26^, the rise time is important whereas; for high repetition rate operation, the decay time should be considered.

**Table 4:** Detector output—deposition angle curve after TEA-CO_2_ laser pulse illumination.

Deposition angle (degree)	Output signal (V)
30	4.20
40	5.20
50	6.00
60	12.2
70	14.0

**Figure 13:**
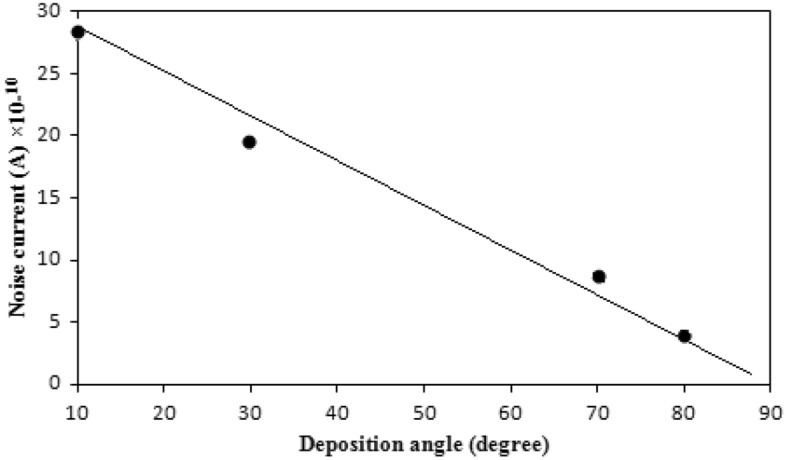
Noise current of the detector versus angle of deposition.

The output generated signal (Volt) across the obliquely deposited film is given as^27^

15 $$ V = \mathop \int \limits_{T1}^{T2} \mathop \alpha \limits^{\prime } \Delta T $$

where ά is the thermo-electric power coefficient, and $\Delta T$ is the temperature rise due to laser heating. The output signal from these detectors will increase with a higher thermoelectric coefficient and temperature rise. In addition, when exposed to a high- intensity laser pulse, the columnar structure that forms in the film, as a result of oblique angle deposition, is essential for producing $\Delta$T. The operation of the detector, which depends on its dark current, is affected by the noise current^28–30^. As seen in Fig. 13, the noise current decreases at large deposition angles. This is as a result of the reduced noise current caused by the increased electrical resistivity brought on by columnar crystalline growth and the development of anisotropic strains in the thin layer.

**Figure 14:**
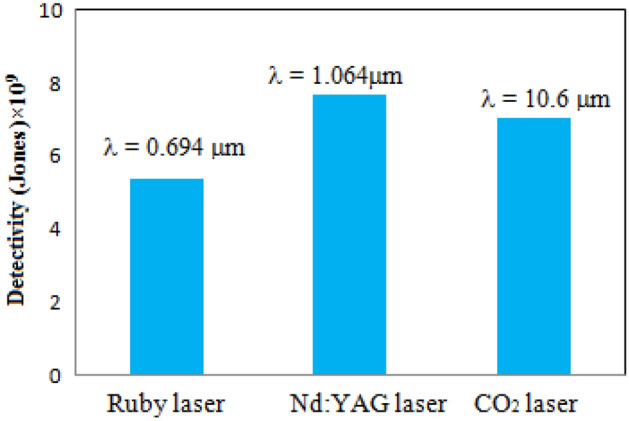
Histogram of detectivity of the detector prepared at θ = 70° as a function of laser wavelength at zero bias.

Figure 14 shows the specific detectivity variation with wavelength at room temperature. Particularly in the IR band, the specific detectivity (D*) of the detector is a crucial figure of merit. It can be calculated using the relationship shown below:

16 $$ D^{*} = \frac{{\left( {A\Delta f} \right)^{0.5} }}{{I_{n} }} $$

where A is the detector’s area, Δf is the frequency bandwidth, and I_n_ is the noise current. The noise current is a summation of three terms: the thermal generation noise, the background noise and the shot noise. For our detectors, the shot noise (dark current I_d_) is predominant source of the noise current. The noise current is related to the dark current by the following equation:

17 $$ I_{n} = \sqrt {2e\Delta fI_{d} } $$

where e is the electron charge.

Table 5 shows the significant parameters of the antimony detector (present work) as compared to common types of IR detectors^31,32^. The antimony detector has a very comparable detectivity to PdSe_2_-MoSe_2_ heterostructure detector; despite the very large area of the antimony detector.

**Table 5:** A comparison of present work (antimony detector parameters) with commercial IR detectors^31,32^.

Detector type	Bias voltage (V)	T_op_ (k)	D* (Jones) at 10.6 µm	Area	Operating spectral region (µm)
PtSe_2_	1	300	10^9^	Small	0.4–10.6
PdSe_2_–MoSe_2_	1	300	8.21 × 10^9^	Small	0.4–10.6
PdSe_2_	1	300	10^9^	Small	0.4–10.6
HgCdTe	1	300	1.5 × 10^7^	Small	0.4–10.6
This work	zero	300	7 × 10^9^	Large	0.694–10.6

**Figure 15:**
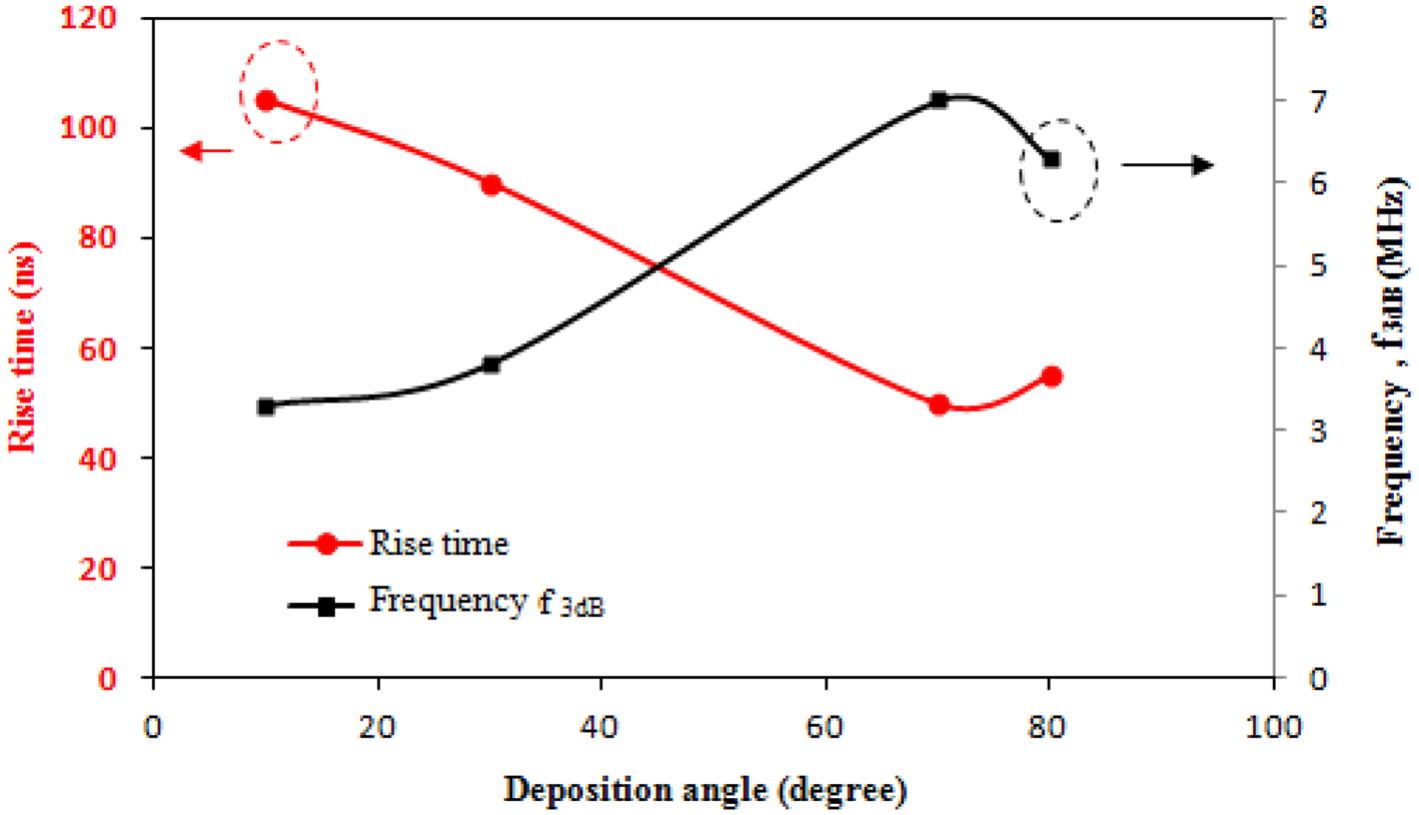
Variation of rise time and frequency bandwidth of the Sb detector with deposition angle for CO_2_ laser pulse without using external bias.

The current antimony detectors display roughly the same specific detectivity for the 1.064–10.6 µm bands as heat detectors. This is mostly attributable to the output voltage's significant dependency on the temperature rise caused by the laser heating, ΔT, as opposed to semiconductors' energy gaps. The current antimony detectors have a detectivity that greater than that of the widely used uncooled HgCdTe detector for CO_2_ laser pulse detection^33^. This is due to the large noise current of HgCdTe photodetector at room temperature, which is a result of its small energy gap^34^. The noise equivalent power of the antimony detectors is around 3.6nW, which is the primary factor in attaining a high D* at room temperature. Figure 15 depicts a linearly descending rise time and an increasing frequency bandwidth (f_3dB_) with a deposition angle that reaches 50 ns and 3.1 MHz at a 70° angle. The fast temperature gradient (∂T/∂t) produced by the columnar structure between the detector terminals is responsible for the antimony detectors' quicker rise times. The rise time of the Sb detector in the current study was marginally quicker than the decay time, which indicates a slower cooling rate (depending on substrate) than the laser heating rate. A balanced amount of heating and cooling must be achieved when choosing the substrate in order to obtain a symmetrical laser pulse from the detector.

## Conclusion

The current study has successfully completed its intended objective, which included the fabrication of self-powered, room-temperature, low-noise, fast, and highly responsive broad-band antimony detectors. The fabricated detectors exhibited good linearity characteristics; indicating their recommended use for laser energy measurements. Besides; the low wettability of these detectors will recommend them for use in open-air applications, without environmental protection. These detectors also have a very high damage threshold to laser pulses, a very large area, and an extremely inexpensive cost. This research project fits into the framework of results on the fabrication of laser detectors that have been published. At room temperature, the studied antimony detectors had (10^9^ Jones) detectivity and 50 ns rise time. To the best of our knowledge, the findings are genuine and will revolutionize detector technology; particularly when it comes to picking up mid-infrared short laser pulses.

## Supplementary Information

Supplementary Information 1.

Supplementary Information 2.

Supplementary Information 3.

Supplementary Information 4.

## Data Availability

The datasets generated and/or analyzed during the current study are available from the corresponding author (R. Ismail) on reasonable request.
