# Supplementary figures and images for: Ultimate figures of merit broadband self-powered obliquely deposited antimony thin film laser detectors
Source: Sci Rep. 2022 Nov 17;12:19794. doi: 10.1038/s41598-022-24116-6 (PMC9672117; doi:10.1038/s41598-022-24116-6)

**
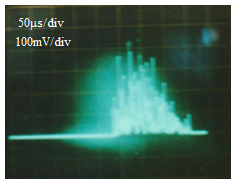
**

**Fig. S1: Ruby laser pulse recorded by Sb detector deposited at θ =70°**

Supplement: Supplementary file 1 — Supplementary Information 1. [file 41598_2022_24116_MOESM1_ESM.docx]

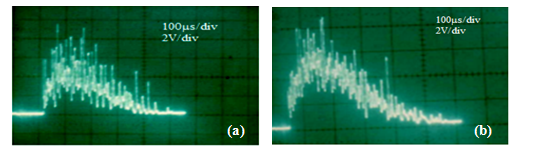


**Fig. S2: Nd: YAG output laser signal from the antimony detectors at two thicknesses (a) 200nm and (b) 300 nm**

Supplement: Supplementary file 2 — Supplementary Information 2. [file 41598_2022_24116_MOESM2_ESM.docx]

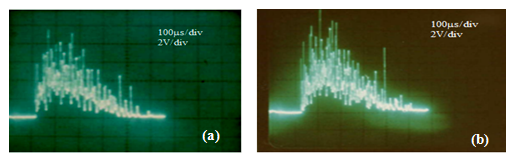


**Fig. S3: Nd: YAG output signal from the antimony detectors at two laser energies (a) 20 mJ and (b) 40 mJ**

Supplement: Supplementary file 3 — Supplementary Information 3. [file 41598_2022_24116_MOESM3_ESM.docx]
