# Supplementary material for: Ultimate figures of merit broadband self-powered obliquely deposited antimony thin film laser detectors
Source: Sci Rep. 2022 Nov 17;12:19794. doi: 10.1038/s41598-022-24116-6 (PMC9672117; doi:10.1038/s41598-022-24116-6)
Supplement: Supplementary file 4 — Supplementary Information 4. [file 41598_2022_24116_MOESM4_ESM.docx]

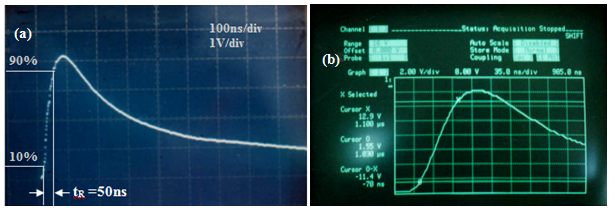


**Fig. S4: TEA-CO_2_ laser signals captured by antimony detector deposited with 70^o^ with thickness of (a) 200 nm and (b) 300 nm**
